# Case Report: Chagas Disease in a Traveler Who Developed Esophageal Involvement Decades after Acute Infection

**DOI:** 10.4269/ajtmh.22-0461

**Published:** 2023-01-16

**Authors:** Norman L. Beatty, Rodrigo F. Alcala, Nelson A. Luque, Mark Radetic, Priti Joshi-Guske, Eyad Alakrad, Colin J. Forsyth, Davidson H. Hamer

**Affiliations:** ^1^ Division of Infectious Diseases and Global Medicine, Department of Medicine, University of Florida College of Medicine, Gainesville, Florida;; ^2^ Emerging Pathogens Institute, University of Florida, Gainesville, Florida;; ^3^ Division of Gastroenterology, Hepatology & Nutrition, Department of Medicine, University of Florida College of Medicine, Gainesville, Florida;; ^4^ Drugs for Neglected Diseases Initiative, New York, New York;; ^5^ Department of Global Health, Boston University School of Public Health, Boston, Massachusetts;; ^6^ Section of Infectious Diseases, Department of Medicine, Boston University School of Medicine, Boston, Massachusetts;; ^7^ Center for Emerging Infectious Disease Research and Policy, Boston University, Boston, Massachusetts;; ^8^ National Emerging Infectious Disease Laboratory, Boston University, Boston, Massachusetts

## Abstract

Travelers to Chagas disease endemic regions of Latin America may be at risk for *Trypanosoma cruzi* infection. We report a 67-year-old woman who screened positive for *T. cruzi* infection while donating blood. The patient had a history of an unusual febrile illness and marked swelling of the face sustained at age 10 after camping in northern Mexico that led to a 3-week hospitalization without a diagnosis. More than 4 decades later, rapid diagnostic tests and commercial and confirmatory serology for Chagas disease were all positive for *T. cruzi* infection. On evaluation, the patient described a progressive chronic cough, gastroesophageal reflux, and dysphagia for > 10 years. There was no evidence of any cardiac complications. However, esophageal manometry demonstrated significant dysmotility, with 90% of swallows being ineffective with evidence of esophageal pressurization and retrograde peristalsis in several swallows, suggesting early autonomic disruption due to Chagas disease esophagopathy. In this report, we highlight the importance of travel-related Chagas disease among travelers to endemic regions and the need to further identify potential risks of transmission among this at-risk population.

## INTRODUCTION

Chagas disease (CD) is a neglected tropical disease endemic in 21 Latin American countries throughout Central and South America.^1^ Travelers who visit regions with high levels of endemic CD, especially with the active presence of vectors and oral transmission, could be exposed to *Trypanosoma cruzi* infection depending on their itinerary, purpose of travel, and activities, but very little is known about the actual risk among this population. According to the CDC’s *Yellow Book 2020: Health Information for International Travel*, the risk for CD is extremely low but increases if staying in poor-quality housing or ingesting food or drink products potentially contaminated with the parasite.^2^ Travel-related CD has been reported in the United States,^3^ Canada,^4^ and certain European countries^5^ but is considered a rare event. Domestic travelers within Latin America have also been found to be at risk for acquiring CD via travel-associated activities such as accidental oral consumption of the parasite.^6^ We present a unique case of a traveler who developed CD with esophageal involvement decades after acute infection acquired while visiting a CD endemic region of Mexico. Transmission of CD among those visiting endemic regions is not well understood, and this report aims to bring further awareness of this risk among travelers.

## CASE REPORT

A 67-year-old female with a past medical history of hypertension, hyperlipidemia, invasive ductal breast carcinoma with lumpectomy treated with aromatase inhibitor and localized intensity-modulated radiation therapy (IMRT) to the left breast (2016), left eye retinal detachment with partial blindness, gastroesophageal reflux disease (GERD), chronic dry cough, and progressive dysphagia presented to our infectious diseases clinic for evaluation of positive *T. cruzi* infection screening assays after routine blood donation in September 2020. She had never donated blood in the past. A complete review of systems revealed a > 10-year history of GERD with a progressively worsening dry cough that had led to coughing spells and subsequent left eye retinal detachment from extreme coughing. Investigations performed by several ear, nose, and throat specialists did not reveal the etiology of the cough, but it was thought to be from GERD. She began to develop difficulty swallowing liquids during the last 2 years along with transient sensations of food getting stuck in her esophagus. Proton-pump inhibitors and H_2_-blockers helped with reflux symptoms but had no effect on her dysphagia. She denied abdominal pain or bloating and normally had a bowel movement every 2 or 3 days, although she reported bouts of intermittent, chronic constipation (6 or more days between bowel movements) since childhood. Previous colonoscopy performed for routine surveillance of colorectal cancer was normal 2 years prior. She denied any heart palpitations, shortness of breath, substernal chest pain, lower extremity edema, irregular heartbeats, or central nervous system deficits.

The patient was born in Nashville, Tennessee and lived in a suburban setting most of her childhood and early adult life. Her mother was born in Tennessee. The patient has lived in north Florida for approximately the last 20 years in a semi-rural setting and is a retired elementary school teacher. She has not lived anywhere else in the United States. The patient denied blood transfusions in her lifetime and is not an organ donor recipient. She denied any known exposure to the triatomine vector (kissing bug) while living in Tennessee or Florida, after being shown several species of naturally occurring *Triatoma* preserved in resin and images from entomological keys. She did not recall ingestion of any uncooked or undercooked wild game meat and is not a hunter. She has never lived in Latin America but, after a detailed history, she described an acute febrile illness sustained at age 10 that occurred during a family camping trip in 1964. The camping trip took place in the state of Nuevo León in northern Mexico near the city of Monterrey. The family slept in pop-up tents and outdoors on the ground within a national park. She recalled being bitten by insects while camping and having welts on her body. While driving back to Tennessee, she developed high-grade fever and bilateral facial swelling. She was admitted to a university hospital in Nashville, Tennessee for 3 weeks and was treated with antibiotics. Eventually her condition improved, and the facial swelling slowly resolved. She was discharged home without a clear explanation of the etiology of her febrile illness. No other family members on the trip developed an illness. Hospital medical records from this event were not available from that time period.

On physical examination her vitals were normal. There were no abnormal findings in the oral cavity. Her lungs were clear to auscultation, and her heart rhythm was regular with normal S1 and S2 without murmur. Her abdomen was soft, nondistended, and with normal bowel sounds. Her lower extremities were without edema and the skin was without rashes. Cranial nerves were grossly intact, and no neurological deficits were noted. Rapid diagnostic testing to investigate for CD was performed in the clinic following each manufacturer’s instructions (Chagas Detect™ Plus; InBios International, Inc., Seattle, WA; DPP^®^ Chagas system; Chembio Diagnostic Systems, Inc., Hauppauge, NY) and was found to be reactive. Serum samples sent to two commercial reference laboratories (Weiner Chagatest ELISA recombinante v. 3.0, Quest Diagnostics laboratory, San Juan Capistrano, CA; Hemagen Chagas kit EIA, Associated Regional and University Pathologists laboratory, Salt Lake City, UT) were both reactive. Confirmatory serological testing performed at the CDC was positive for *T. cruzi* anti-IgG (Weiner Chagatest ELISA recombinante v. 3.0) and trypomastigote excretory-secretory antigen by immunoblot. Baseline complete blood counts and metabolic panel were within normal limits. HIV screening and *Strongyloides stercoralis* IgG were negative.

**Figure 1.**
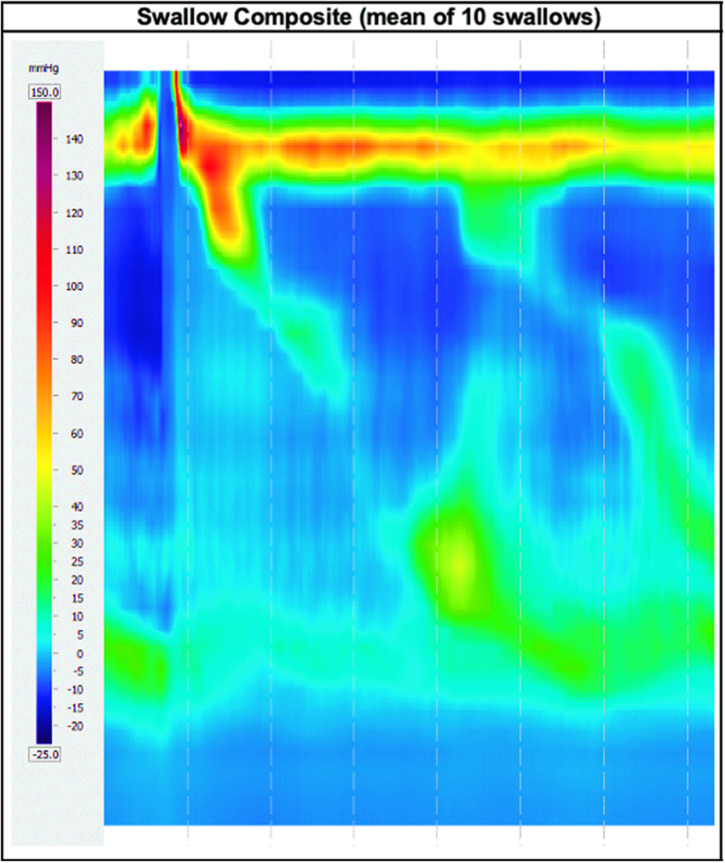
High-resolution esophageal manometry findings demonstrating ineffective esophageal motility with weak/failed propagation of the normal esophageal pressure wave during deglutition.

Further workup took place investigating both the cardiac and gastrointestinal organ systems. Chest X-ray, electrocardiogram, and transthoracic echocardiogram did not reveal any abnormalities. Cardiac magnetic resonance imaging was also performed and did not reveal any radiologic evidence of Chagasic cardiomyopathy. Esophagogastroduodenoscopy was unremarkable, with biopsies of the esophagus, stomach, and duodenum demonstrating only mild chronic gastritis with negative *Helicobacter pylori* testing. High-resolution esophageal manometry (HREM) revealed that 90% of swallows were ineffective, with 60% failed swallows and 30% weak swallows (Figure 1). Although the esophagogastric junction (EGJ) demonstrated normal relaxation, the median integrated relaxation pressure (IRP) was in the upper limit of normal (12.6 mm Hg, normal < 15 mm Hg). The combination of 90% ineffective swallows with borderline normal median IRP was highly suggestive of an evolving achalasia or achalasia phenotype secondary to esophageal involvement of CD. Impedance analysis demonstrated complete bolus clearance in 60% of the swallows. Upper esophageal sphincter had normal tone, relaxation, and residual pressures. Multiple rapid swallow testing indicated a good peristaltic reserve, and the rapid drink challenge yielded complete EGJ/deglutitive inhibition.

A diagnosis of ineffective esophageal motility (IEM) (based on Chicago classification version 4.0^©^ for high-resolution manometry^7^), with some swallows showing esophageal pressurization and retrograde peristalsis, was made. Risks and benefits of antiparasitic treatment were discussed with the patient, who decided to undergo standard antitrypanosomal therapy and was treated with oral benznidazole (300 mg) daily divided into two separate doses for a total of 60 days. She tolerated the complete treatment and experienced fatigue, subjective low-grade fever, and headache that self-resolved after finishing the course. The patient did not develop any other systemic adverse effects such as urticarial rash. Weekly complete blood counts and metabolic panels were followed during treatment and yielded mild lymphocytic leukopenia (normal absolute lymphocyte count of 1.0–3.20 × 10^3^/μL; while on benznidazole therapy, the count was 0.36–0.97 × 10^3^/μL) and acute kidney injury (baseline creatinine was 0.9 mg/dL; while on benznidazole therapy, it was 1.09–1.37 mg/dL) that did not require cessation of benznidazole. These laboratory abnormalities self-resolved after the benznidazole treatment course. Approximately 6 months after completing treatment with oral benznidazole, the patient had not noticed any improvement in her dysphagia, but she also thinks that it has not worsened. The patient has been advised on dietary techniques and some restrictions for certain foods to avoid impaction. Other targeted treatments such as surgical intervention and possibly pharmacotherapy will be considered in the future if IEM progresses to achalasia. The patient is being followed up with infectious diseases and gastroenterology annually, including clinical monitoring of esophageal disease and possible colonic involvement.

## DISCUSSION

The risks for CD among travelers residing in the United States after visiting endemic regions of Latin America have been described but are not well characterized. A traveler who spent 3 weeks in Puerto Viejo, Costa Rica developed acute infection 2 days after her trip.^3^ She stayed in a cottage with wooden plank floors under a mosquito net, but a known exposure to the triatomine vector was not mentioned. She was found to have clear evidence of unilateral periorbital edema (Romaña’s sign), nonblanching rash, and one enlarged non-tender cervical lymph node. Her peripheral blood smear showed circulating *T. cruzi* trypomastigotes, and she was treated with the antitrypanosomal agent nifurtimox. Another case reported by the GeoSentinel network in a Canadian woman was described in 2008 after she visited rural regions of southern Mexico for 5 months.^4^ In 2021, the U.S. Chagas Diagnostic Working Group provided recommendations for screening at-risk populations for CD. Travelers who have stayed in an endemic region with confirmed or suspected triatomine exposure (bite or being found within human dwelling), or have slept in a home constructed of mud, adobe, or a thatched roof, or consumed raw or unpasteurized foods/drinks that could be contaminated with *T. cruzi* can be considered for screening with a conditional recommendation based on low-quality evidence.^8^ Travelers are advised to avoid ingesting certain fruit juices previously implicated in acute CD such as guava juice, bacaba, babaçu and palm wine, açai pulp, and raw sugar cane juice.^9^

Our patient developed chronic CD with esophageal involvement ∼40 years after her acute infection while visiting rural regions of northern Mexico. The case is notable because the gastrointestinal involvement of CD is more commonly reported among those infected in the Southern Cone of South America, including regions within the Gran Chaco.^10^^,^^11^ Certain *T. cruzi* discrete typing units (DTUs) are found in the Southern Cone and are known to cause gastrointestinal disease, including DTUs Tc II, Tc V, and Tc VI.^10^^,^^11^ However, as demonstrated in this case and other cases from Mexico, gastrointestinal disease can be found in endemic regions where TcI is the dominant DTU causing human disease.^12^^–^^16^ One seroprevalence study from the state of Puebla, Mexico revealed 16.9% (*n* = 12/71) of those with CD had gastrointestinal symptoms, including 4 patients with esophageal manometry abnormalities.^15^ Chagas disease esophagopathy (CDE) more commonly evolves into achalasia characterized by aperistalsis with EGJ outflow obstruction (abnormal median IRP). However, those with CDE often present with varying degrees of esophageal dysmotility seen on HREM, which is likely related to the evolution of autonomic dysregulation seen in CDE.^16^^–^^21^ One investigation of 62 patients confirmed with chronic CD living in Spain found that 22.6% (*n* = 14/62) had pathologic HREM findings consistent with CDE prior to antitrypanosomal treatment.^22^ Most (*n* = 13/14) were diagnosed with IEM, and 1 patient was diagnosed with fragmented peristalsis.^22^ Another study in Spain revealed that 8.8% (*n* = 6/67) with chronic CD had IEM after esophageal manometric testing.^23^ Dysphagia is the most common and first symptom seen in CDE but may not always correlate to the degree of megaesophagus seen radiologically.^17^^–^^23^ Regurgitation, weight loss, dyspepsia, increased thirst, chest discomfort, and cough are all associated symptoms of CDE, so a thorough review of symptoms should be undertaken in all those with serological evidence of CD.^17^^–^^23^ One limitation of this case report is that our patient did receive a short course (6 weeks; ≈50 Gy) of IMRT for 0.4-cm invasive ductal carcinoma (TIaN0M0) in 2016 after lumpectomy. She did not experience acute radiation-induced esophagitis during this treatment or receive concomitant chemotherapy, which has been described in those who develop late-onset esophageal toxicity.^24^ Limited data exist (if any) with regard to HREM findings and those with a previous history of localized IMRT to a single breast.^24^ IMRT is a specialized technique that uses small photon and proton beams to precisely deliver radiation to the tumor or lumpectomy cavity to avoid or reduce exposure to surrounding tissues.^25^ Late complications of IMRT are typically described locally in the breast and do not extend into deeper tissues found in the thorax.^25^^–^^27^ Because late complications in the esophagus after receiving IMRT have not been reported, and her symptoms had already started prior to her diagnosis of left breast cancer, we think that the current clinical presentation supports CD with esophageal involvement.

In conclusion, CD among those traveling to endemic regions is likely a rare event. Our case highlights the importance of keeping CD in the differential diagnosis for travelers who may be manifesting clinical evidence of chronic disease, even decades after their return.
